# Individual risk-taking behaviour in black-capped chickadees (*Poecile atricapillus*) does not predict annual survival

**DOI:** 10.1098/rsos.220299

**Published:** 2022-07-27

**Authors:** Kimberley J. Mathot, Josue D. Arteaga-Torres, Jan J. Wijmenga

**Affiliations:** ^1^ Department of Biological Sciences, University of Alberta, Edmonton, Alberta, Canada T6G 2E9; ^2^ Canada Research Chair in Integrative Ecology, University of Alberta, Edmonton, Alberta, Canada T6G 2E9

**Keywords:** animal personality, risk-taking, survival, foraging behaviour

## Abstract

Within species, individuals often show repeatable differences in behaviours, called ‘animal personality’. One behaviour that has been widely studied is how quickly an individual resumes feeding after a disturbance, referred to as boldness or risk-taking. Depending on the mechanism(s) shaping risk-taking behaviour, risk-taking could be positively, negatively, or not associated with differences in overall survival. We studied risk-taking and survival in a population of free-living black-capped chickadees (*Poecile atricapillus*) in which we previously showed repeatable among-individual differences in risk-taking over the course of several months. We found no evidence that variation in risk-taking is associated with differences in annual survival. We suggest that variation in risk-taking is likely shaped by multiple mechanisms simultaneously, such that the net effect on survival is small or null. For example, among-individual differences in energy demand may favour greater risk-taking without imposing an overall mortality cost if higher energy demand covaries with escape flight performance. We propose directions for future work, including using a multi-trait, multi-year approach to study risk-taking, to allow for stronger inferences regarding the mechanisms shaping these behavioural decisions.

## 1. Introduction

Within populations, individuals often exhibit repeatable differences in behaviour, referred to as animal personality [1]. For example, within populations, individuals vary consistently in how quickly they return to baseline levels of activities following manipulations of perceived predation risk (e.g. [2–7]). This behaviour is often referred to as ‘boldness’ or ‘risk-taking’. Understanding the causes and consequences of animal personality is a major area of research in contemporary behavioural and evolutionary ecology and models of adaptive animal personality variation often invoke state-dependent behavioural decisions and/or trade-offs to explain the maintenance of such variation within populations [8–11].

Among-individual differences in risk-taking may arise due to trade-offs and/or state-dependent payoffs. For example, responsiveness to predators outside of the breeding season may trade off different components of mortality risk; risk of mortality due to starvation versus risk-of mortality due to predation such that variation in risk-taking results in similar overall survival. However, the resolution of these trade-offs may also be shaped by an individual's state, in which case, differences in risk-taking may be associated with differences in fitness. Individuals with intrinsically lower vulnerability to predators may behave more boldly in response to predators and therefore increase resource acquisition without incurring higher risks of mortality due to predation [12]. For example, in prey with gape-limited predators, larger individuals may be intrinsically less vulnerable [12], while in birds, individuals with larger pectoral muscles may be less vulnerable due to greater escape flight performance [13]. In this case, higher risk-taking would be associated with higher survival. On the other hand, individuals with intrinsically lower risk of starvation (e.g. low metabolic rates) may exhibit relatively low boldness compared to individuals with intrinsically higher risk of starvation (e.g. high metabolic rates) to achieve similar realized risk of starvation, while at the same time having lower risk of mortality due to predation [6]. Given that the consequences of boldness for survival are likely to depend not only on context (e.g. food and/or predator abundance), but also depend on aspects of an individual's state (e.g. body condition, ability to evade predators, etc.), it is perhaps not surprising that studies that have attempted to evaluate survival consequences of among-individual differences in boldness or risk-taking have yielded mixed results [14–16].

In an earlier study, we showed that response to manipulations of perceived predation risk in free-living black-capped chickadees (*Poecile atricapillus*) vary markedly both within and among individuals [2]. Within individuals, variation in risk-taking was shaped by ambient temperatures, with lower temperature generally favouring shorter latencies to resume feeding [2]. This effect was presumably because lower temperatures (below thermoneutrality) increase energy expenditure in small birds [17], favouring faster resumption of feeding behaviour. Individuals also exhibited repeatable differences in risk-taking. After experimental manipulations of perceived predation risk, some individuals resumed feeding within minutes, while others resumed feeding after hours, and these among-individual differences were repeatable for at least several months over the non-breeding season [2]. Here, we evaluate whether among-individual differences in response to experimental manipulations of perceived predation risk are associated with differences in feeding rates and annual survival and evaluate support for the three non-exclusive mechanisms outlined above: (i) differences in allocation to starvation versus predation avoidance, (ii) differences in intrinsic vulnerability to predation and (iii) differences in intrinsic vulnerability to starvation. To do this, we quantified foraging and risk-taking in 79 black-capped chickadees over the course of a winter, and subsequently tracked their survival to the following winter. We include feeding rates prior to the experimental manipulations of perceived predation risk because foraging can also be viewed as a form of risk-taking in that it provides access to food resources while exposing individuals to greater risk [18]. Thus, we expected feeding rates and latency to resume feeding following a disturbance to be negatively correlated (i.e. higher feeding rates associated with shorter latencies). Further, if these are both expressions of risk-taking, then higher feeding rates and shorter latencies to resume feeding should have similar survival consequences.

## 2. Methods

### 2.1. Study site and study population

This study was carried out in a marked population of black-capped chickadees at the University of Alberta Botanic Garden (UABG) in Devon, Alberta, Canada (53 °2 402 700 N, 113 °4 504 100 W). The study area is 97 hectares: 32 hectares of display gardens and 65 hectares of mixed forest. A marked population of black-capped chickadees was established in the fall of 2017 and catching and banding are conducted annually to maintain the marked population. Chickadees were caught using mist-nets placed near feeders. Upon capture, any birds that had not previously been caught were fitted with Canadian Wildlife Service aluminium bands for unique identification. Two short-behavioural assays were carried out as part of another study totally less than 4 min; a field cage exploration test (following the protocol outlined in [19]) and handling aggression tests (following the protocol outlined in [20]). Following these two tests, standard morphometric data were collected (e.g. tarsus, bill length, bill depth, wing length), body mass was recorded, and a small blood sample was collected from the brachial vein to allow for molecular sexing [21]. As part of another study aimed at assessing the effects of passive integrated transponder (PIT) tags and methods on chickadees, from 2017 to 2019, birds were randomly assigned to receive no PIT tag, a PIT tag attached a colour band, or a PIT tag implanted subcutaneously [22]. Because we relied on radio frequency identification (RFID) to detect birds at feeders (see below), the data presented here are only for chickadees that were fitted with leg band PIT tags, as the implanted PIT tags were found to have unreliable detectability (see [2] for further details). Comparisons of observations of 353 feeder visits by chickadees with leg band embedded PIT tags from video recordings against RFID registrations confirmed that the leg band embedded PIT tags were registered with 100% reliability [2].

### 2.2. Foraging and risk-taking behaviour

We analysed within- and among-individual differences in both foraging (i.e. visit rates to feeders prior to a disturbance) and risk-taking (i.e. latency to resume feeding following a disturbance). Within-individual changes (i.e. behavioural plasticity) refer to changes in behaviour expressed by the same individual in different instances, while among-individual differences refer to differences in the average behaviour expressed by different individuals. By analysing both feeding rates and latency to resume feeding following a disturbance, we were able to assess whether associations between risky behaviours and survival were generalizable, or context-dependent (i.e. whether they depend on the degree of perceived risk).

Foraging behaviour and risk-taking were assessed in experiments that took place between November 2018 and March 2019 at eight feeder locations spread throughout the study site. Feeders were placed at least 270 m apart based on previously reported winter territory sizes from other populations of black-capped chickadees [23], with the aim of providing a single feeder per winter flock (see electronic supplementary material, figure S1). Feeders were filled with black oil sunflower seeds and equipped with an RFID antenna around the feeder opening that is connected to a circuit board with internal clock and SD card for data storage. Details on the feeders and RFID system are provided elsewhere [2], but briefly, when a bird with a leg band embedded PIT tag visits the feeder, its unique transponder code, and the date and time of the visit are registered to the SD card. Throughout the experiments, feeders were visited every 4 days to top up the seeds, to replace the batteries and to collect the data that had been written to the SD cards. These visits to the feeder were always conducted on non-experimental days so that they did not create disturbances that might interfere with interpretation of treatment effects.

Experiments were conducted using a 2 × 2 factorial design using different combinations of acoustic (yes/no) and visual (yes/no) cues of predation resulting in a total of four treatment combinations. The acoustic cue was comprised of mobbing calls of chickadees recorded in another population (located *ca* 40 km from the current study population) produced in response to merlin (*Falco columbarius*) mounts. Eight unique 1 h files were created that were made up of alternating sequences of mobbing calls (ranging from 5 to 20 s in length and comprised of the mobbing calls of between 1 and 4 chickadees, repeated over 1 min periods) and bouts of silence (ranging from 60 to 180 s). Each of the eight unique files included the same range of flock sizes in the mobbing bouts (1–4). The sequence files were played back using portable speakers (Shockwave, FoxPro, Lewistown, PA, USA) that were placed on top of a pole 3 m in front of the feeder. The volume at which the calls were broadcast could be heard up to distances of approximately 80 m (J.D.A.-T., 2018, personal observation). Further details about the recordings are provided in Arteaga-Torres *et al*. [2]. We used six different taxidermic mounts of juvenile merlin as our visual cue of predation risk. During presentation, the mount was carried to the focal feeder in a plastic box and then removed from the box and placed on a pole that was 3 m in front of the feeder (as with the speaker during acoustic treatments) with the mount facing the feeder (see electronic supplementary material, figure S2). The height of the pole was such that the mount lined up with the height of the feeder opening. These mounts were visible at distances ranging from around 20 to 50 m (J.D.A.-T., 2018, personal observation). Merlin were selected as the model predator because they specialize in small birds, including chickadees [24], and are present in the study area throughout the winter (based on records in the eBird digital repository: https://ebird.org/species/merlin). Our control treatments for both the acoustic and visual cues of predation risk were designed to control for the non-biological components of our experimental treatments. As such, the control for the acoustic playback consisted of the presence of the speaker (not broadcasting sound). The control for the visual cue consisted of the presence of the pole placed near the feeder, but without a taxidermic mount. All treatments were 1 h in duration. This treatment length was chosen to allow for sufficient time for birds to experience the treatment, but was intended to be short enough to prevent habituation. Previous analyses found no evidence of habituation by chickadees to the treatments [2].

We used a stratified random design to assign treatments to feeders such that (i) each experimental day a maximum of one treatment was carried out at any given feeder and (ii) each experimental day, each of the four treatment levels was carried out (i.e. one control, one acoustic, one visual and one combined). A complete replicate consisted of each of the four treatments being carried out at each of the eight feeders. Treatment start times were 09:30, 11:00, 12:30 and 14:00. Within each experimental day, the order of the treatments was randomized. To minimize potential carry-over and/or habituation effects of our treatments, we only conducted treatments (experimental days) every second day during any given replicate, with at least 7 days break between successive replicates. This meant that within a given replicate, three 1 h long predator presentations (one visual, one acoustic and one visual + acoustic) plus 1 h control treatment occurred at a single feeder over the course of 15 days (8 days to complete the replicate + 7 days between replicates). We carried out four complete replicates of the experiment at each of the eight feeders.

Only birds that were present at feeders in the 1 h immediately preceding a treatment were included in the analysis. This was done to increase the likelihood that birds detected at a feeder following a given treatment had been in the vicinity of the feeder when the treatment was carried out, and therefore, likely to have experienced the treatment. We used the count of feeder visits in the hour immediately preceding the treatment as a measure of feeding rate (visits/h). We define risk-taking as the latency to resume feeding following the presentation of a treatment at a feeder (i.e. the time in seconds from the start of the treatment to the first visit by each individual to the feeder), with shorter latencies corresponding to greater risk-taking.

### 2.3. Survival data

We monitored detections of the 79 birds (47 males and 32 females) for which we obtained feeding rate and risk-taking measures at RFID equipped feeders over the subsequent 2 years to allow us to estimate annual survival. Chickadees are non-migratory and form stable winter flocks [23]. Surviving birds remain part of the same winter flock in subsequent years, and occupy the same core winter territory [23]. As such, we used re-detections in the study area as a proxy for survival. Birds that were not detected in the year after the collection of the behavioural data (*N* = 35) were also not detected in the following year, suggesting that they were not simply transiently absent from the study area. We used detection (yes/no) in the year following collection of behavioural data as our estimate of annual survival.

### 2.4. Data analysis

We analysed the within- and among-individual correlations between foraging rate and latency to resume feeding after manipulations of perceived predation risk-taking using a bivariate mixed effects models using the R package *MCMCglmm* [25] in the R Statistical Environment [26] using the R Studio interface [27]. We constructed a two-trait model with feeding rate (continuous variable), and latency to resume feeding (continuous variable) as response variables. Foraging rate and latency to resume feeding were both log transformed prior to analysis so that model residuals met the assumption of normality. We opted to transform these variables rather than construct models with Poisson error distributions because the latter resulted in models that were overdispersed (results not shown). We included sex as a fixed effect for both foraging rate and latency to resume feeding because in chickadees males are dominant over females [23], which might logically be expected to affect both foraging and risk-taking decisions. We additionally modelled the effect of temperature because it has previously been shown to affect both foraging and risk-taking in our population [2], presumably because temperatures below thermoneutrality increase energy demand. Daily average temperature, obtained from a nearby weather station (Edmonton International Airport Weather Station, 10 KM SE of field site, https://agriculture.alberta.ca/acis), was included as a fixed effect for hourly feeding rates. We only modelled the treatment effect (categorical variable with four levels), and its interaction with temperature, for the latency to resume feeding as the foraging data was collected prior to treatments (which were assigned using a stratified random design) and therefore, logically could not have been affected by treatment. The temperature was centred and standardized prior to analyses. We included individual ID as a random effect, allowing us to quantify the pairwise among-individual relationships between foraging rate, latency to resume feeding, and survival. We did not include feeder ID as a random effect as earlier analyses showed it to be of minor importance [2]. To ensure model convergence, we ran three chains with 103 000 iterations with a burnin of 3000 and a thinning of 100 to generate 1000 estimates. Posterior density plots were inspected visually to ensure proper model mixing and convergence (see electronic supplementary material, figure S3). Results presented in the text below are from a representative chain using a parameter expanded prior. However, we verified that results were robust to modest changes in the prior specifications (result not shown). Results are presented as the mean and 95% credible interval (CrI) of the 1000 estimates from a single chain. The point estimates and 95% CrIs were used to evaluate support for a given effect (see below). We calculated adjusted repeatability following Nakagawa & Schielzeth [28] as: [among-individual variance]/[among-individual variance + residual variance].

Although we initially attempted to include survival as a third trait in the multivariate model to estimate the among-individual covariance between foraging (continuous trait with repeated measures), latency (continuous trait with repeated measures) and survival (binary trait with a single measure per individual), we were unable to achieve good model convergence. This was also true when constructing bivariate models for behaviour (either foraging or latency) and survival. Thus, to quantify the among-individual correlation between behaviour and survival, we instead ran univariate generalized linear models (glms) of survival (yes/no) as a function of the best linear unbiased predictors (BLUPs) of behaviour derived from the bivariate model described above. To account for the uncertainty in BLUPs, we ran each glm of survival 1000 times using an estimate drawn from the distribution of BLUPs for each behavioural trait [29]. The 1000 estimated effects sizes of behaviour on survival were used to derive an estimated effect size and 95% CrI for the relationship between behaviour (foraging rate or latency) on annual survival. We additionally investigated sex-related differences in survival using a general linear model with survival (yes/no) as the response variable and sex as the predictor. All glms with survival as a fixed effect were fitted with a binomial error distribution.

We describe estimates with CrIs that did not overlap zero as providing strong support for an effect, while estimates that were centred on zero are described as providing no support for an effect, or strong support for lack of an effect. For estimates that were not centred on zero, but whose CrIs overlapped zero, we calculated Bayesian *p*-values based on the proportion of counts of estimates that were above or below zero, depending on the direction of the estimated mean. Estimates with Bayesian *p*-values less than 0.15 are referred to as showing moderate support for an effect because this corresponds to more than five times greater support for the interpretation of an effect compared to the interpretation of no effect.

## 3. Results

Of the 79 birds for which we quantified risk-taking behaviour in the winter of 2018/2019, 44 were detected using the feeders the following winter (2019/2020). The 35 birds that were not detected using the feeder the following winter were assumed to have died. This reflects an annual survival rate of approximately 56%.

**Figure 1.**
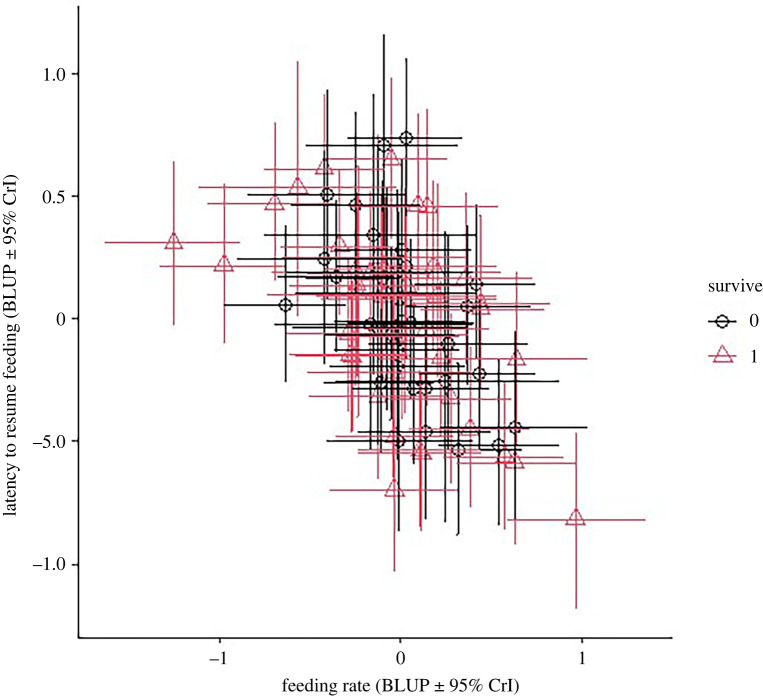
Among-individual correlations between feeding rate, latency to resume feeding after predator cues. Each point represents an individual's mean BLUP derived from the bivariate model. Whiskers denote 95% CrIs. Annual survival outcome is illustrated by colour: survived = black circles, died = red triangles. There is a strong negative correlation between feeding rate and latency to resume feeding, but no evidence that either trait predicts survival outcome.

Feeding rate varied as a function of both temperature and sex (table 1). As temperature increased (i.e. conditions became less energetically challenging), foraging rates decreased (temperature effect: *β* = −0.14, 95% CrI = −0.19, −0.08). Males also exhibited higher feeding rates compared to females (sex effect: *β* = 0.26, 95% CrI = 0.02, 0.47). Further, there was significant among-individual variation in feeding rates (*r* = 0.22, 95% CrI = 0.14, 0.34). Analyses of the response to different cues of predation is presented in detail elsewhere [2]; however, our multivariate models reproduce the key findings of no evidence for sex-related differences in latency to resume feeding, and longer latencies to resume feeding with increased ambient temperatures for most treatments (table 1). As previously reported [2], there was also significant among-individual variation in latency to resume feeding (*r* = 0.22, 95% CrI = 0.15, 0.37). Feeding rate and latency to resume feeding after manipulations of perceived predation risk were negatively correlated within-individuals (*r* = −0.18, 95% CrI = −0.24, −0.12), presumably due to the common effect of ambient temperature. Foraging and latency to resume feeding were also strongly negatively correlated at the among-individual (*r* = −0.50, 95% CrI = −0.76, −0.25, figure 1).

**Table 1.** Sources of variation in feeding rates (feeder visits per hour) prior to manipulations, and latency to resume feeding following manipulation of perceived predation risk (seconds). Estimates derived from MCMCglmm model with log feeding rate and log latency to resume feeding as response variables. See main text for full model details.

fixed effects	log feeding rate (visits per hour)	log latency to resume feeding (seconds)
	*β* (95% CI)	*β* (95% CI)
intercept^a^	−0.16 (−0.34, −0.01)	0.34 (0.15, 0.53)
sex^b^	0.26 (−0.03, 0.48)	−0.01 (−0.22, 0.20)
control treatment^c^	n.a.	−0.70 (−0.85, −0.56)
acoustic treatment^c^	n.a.	−0.56 (−0.69, −0.40)
visual treatment^c^	n.a.	0.06 (−0.06, 0.21)
temperature^d^	−0.14 (−0.19, −0.08)	n.a.
temperature^d^: control	n.a.	0.15 (0.04, 0.24)
temperature^d^: acoustic	n.a.	0.11 (0.01, 0.22)
temperature^d^: visual	n.a.	−0.01 (−0.11, 0.09)
temperature^d^: acoustic + visual	n.a.	0.11 (−0.01, 0.22)
**random effects**	***σ* (95% CI)**	***σ* (95% CI)**
individual ID	0.19 (0.12, 0.28)	0.17 (0.10, 0.25)
residual	0.80 (0.76, 0.87)	0.72 (0.66, 0.78)
**repeatability**	**r (95% CI)**	**r (95% CI)**
individual^e^	0.22 (0.14, 0.34)	0.19 (0.13, 0.26)

^a^Intercept estimated average temperature over study period and for female sex. For latency to resume feeding data, the intercept was estimated during the highest risk treatment (i.e. acoustic + visual).

^b^Sex effect (reference category = female): estimates difference between males and females.

^c^Treatment effects relative to highest risk treatment (i.e. reference category = acoustic plus visual).

^d^Temperature, centred and standardized.

^e^Adjusted repeatability estimated after taking into account fixed effects.

**Figure 2.**
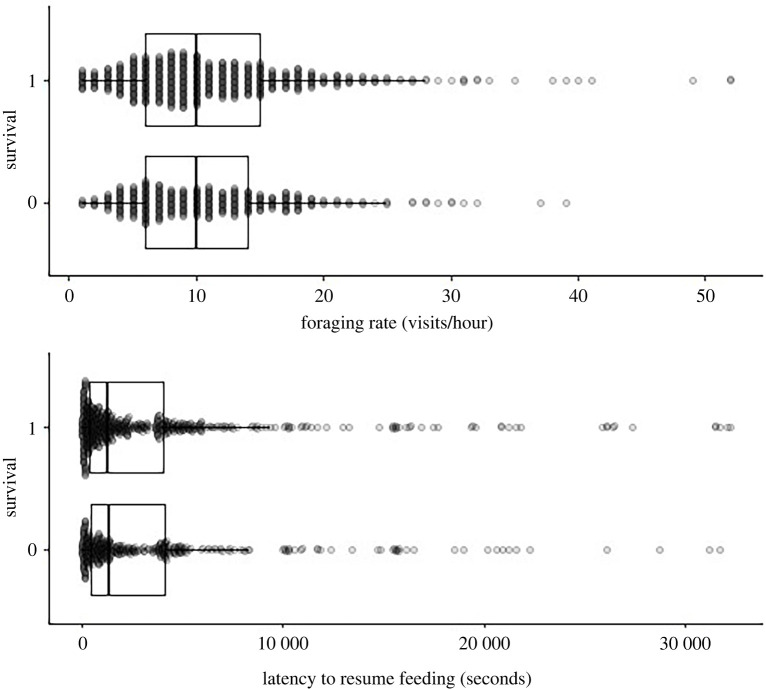
Distribution of foraging rates (*a*) and latency to resume feeding (*b*) as a function of annual survival (0 = no, 1 = yes). Each dot represents an observation, with multiple observations per individual. The outer bounds of the boxes indicate the 25th and 75th interquartile range, and the centre line is the median value. Whiskers denote 10th and 90th percentiles.

We found no support for an effect of either feeding rate (log odds ratio = −0.16, 95% CrI = −0.86, 0.38) or latency to resume feeding (log odds ratio = −0.07, 95% CrI = −0.86, 0.38) on annual survival (figures 1 and 2). Although both point estimates were negative, the confidence intervals overlapped zero substantially (foraging: Bayesian *p*-value = 0.33; latency: Bayesian *p*-value = 0.40), indicating no support for an effect. There was moderate support for a sex effect on survival (Bayesian *p*-value = 0.10), with males (log odds ratio = 0.48, 95% CrI = −0.10, 1.08) tending to have higher annual survival compared with females (log odds ratio = −0.13, 95% CrI = −0.83, 0.57).

## 4. Discussion

We previously showed repeatable among-individual differences in the latency to resume feeding after exposure to predator cues (risk-taking) in the same population of chickadees [2]. Here, we asked whether this variation in risk-taking was associated with variation in foraging rates and annual survival to evaluate support for three potential non-exclusive mechanisms underlying variation in risk-taking: differences in allocation to starvation versus predation avoidance, differences in vulnerability to starvation, and/or differences in vulnerability to predation. Although latency to resume feeding and foraging were strongly negatively correlated both within and among individuals, there was no support for a correlation between either risk-taking or foraging on annual survival. Below, we discuss the implications and limitations of these results, and provide directions for future work.

Both foraging and risk-taking allow individuals to acquire resources while exposing them to risk [18]. Therefore, we expected to observe a correlation between these two traits, which we did. Individuals that had higher foraging rates on average in the observation period prior to our predator treatments also had shorter latencies to resume feeding following predator treatments. We found no support for the interpretation that among-individual differences in either foraging or risk-taking were associated with variation in annual survival. Studies that quantify the relationships between behaviour and survival at the among-individual level, rather than at the phenotypic level, are relatively rare [30]. Nonetheless, our result is in line with two recent meta-analyses that broadly find little support that among-individual differences in foraging behaviour [14], or risk-taking [14,15], are associated with predictable differences in survival.

One explanation for our finding that neither foraging or risk-taking are associated with differences in overall survival is that variation in foraging and risk-taking reflects differences in allocation to avoidance of starvation versus predation. For example, individuals that invest more in starvation avoidance have higher feeding rates and shorter latencies to resume feeding on average, but this does not translate to a net survival benefit because it comes at the cost of increased mortality due to predation. Proper evaluation of this possibility would require data on the sources of mortality, which we do not have. However, we suggest it is unlikely that this can fully account for our results. There is ample evidence that among-individual differences in state variables shape foraging and risk-taking. For example, across a range of taxa, higher energetic needs and/or lower nutritional status are associated with increased foraging and risk-taking [31,32]. We show that within-individual variation in foraging and risk-taking in our population are shaped by within-individual variation in energetic needs, as inferred from ambient temperature. Given that among-individual differences in energetic needs are near ubiquitous in animals [33], it seems likely that among-individual variation in energetic needs would similarly shape among-individual differences in foraging and risk-taking in our population. Such state-dependent behaviour often results in individuals achieving different fitness outcomes [11], yet we found no effect of variation in either foraging or risk-taking on annual survival. How can this be?

We suggest that the lack of relationship between either foraging or risk-taking and annual survival may arise because the expression of these behaviours is shaped by multiple state-dependent mechanisms simultaneously. For example, if vulnerability to predation and vulnerability to starvation act together to shape the expression of foraging and risk-taking behaviour, we may not expect a net effect of either behaviour on overall survival. This is because under among-individual differences in vulnerability to predation (e.g. due to differences in feather condition, flight muscle size, etc.), increased foraging or risk-taking would be associated with lower risk of starvation, while under among-individual differences in starvation (e.g. due to differences in metabolic rate, priority access to feeders, etc.) it would be associated with increased risk-of mortality due to predation. This could occur if vulnerability to predation and energy requirements were themselves linked, for example, if individuals with higher energy requirements also have greater ability to evade predators. Such a pattern would be predicted under the ‘performance’ model of metabolism, whereby individuals with higher energetic requirements are also able to exhibit higher expression of performance related traits such as escape behaviour [34]. Although there is general support for performance models in various animal taxa [31], the relationship has yet to be tested in this system. Nonetheless, at least one study provides some suggestion that this may be the case. In another population of chickadees, individuals that carried more fat also had larger pectoral muscles [35]. Fat stores are sometimes maintained as a buffer against starvation risk associated with high energy requirements [36], and pectoral muscle is important for powering rapid escape flights [13]. Therefore, the correlation between fat stores and pectoral muscle mass reported previously is suggestive of a potential correlation between energy demand and vulnerability to predation, though this is speculative, and requires further testing.

We also observed trait-specific sex effects. Males had significant higher feeding rates and higher annual survival compared to females, but there was no evidence of sex-related differences in latency to resume feeding following a manipulation of perceived risk. These results can be understood in light of the fact that in black-capped chickadees, males are structurally larger and are dominant over females [23,37]. Males are therefore expected to have higher energy requirements (due to their large size) but are also expected to be able to maintain priority access to the best feeding sites. Higher dominance is associated with higher survival in small birds in winter in general [38], and previous studies in black-capped chickadees have also reported higher survival in males [39,40]. If latency to resume feeding after a manipulation of perceived risk was solely due to differences in energy requirements, then we would predict males to return sooner than females. The fact that they did not is consistent with previous work showing that subordinates take relatively more risk in resuming feeding following encounters with predators to offset their generally lower access to food [36,41]. The simultaneous effect of high energy demands in males and low priority access to food in females may lead to a lack of overall sex-effect on latency to resume feeding.

Taken together, our results show that foraging behaviour and risk-taking share common drivers (e.g. temperature), but can also vary independently (e.g. sex affected foraging but not risk-taking). Although we found no support for the interpretation that either foraging rate or latency to resume feeding were associated with differences in annual survival (after controlling for sex effects), we suggest that this pattern may reflect the net effect of multiple mechanisms shaping the expression of foraging and risk-taking, and their relationship to annual survival simultaneously. Many models for adaptive animal personality are based on state-dependence and predict non-equal fitness outcomes for different behavioural types [8,11]. We suggest that it will often be the case that the expression of a given behaviour is simultaneously shaped by multiple state variables whose fitness consequences may cancel out. Studies that simultaneously consider multiple traits are needed to tease apart the relative contributions of different mechanisms. For example, future studies of among-individual differences in risk-taking could aim to quantify individual state variables related to energy requirements and vulnerability to predation, simultaneously. In birds, this could mean measuring metabolic rates and escape flight performance, respectively. Additionally, extending studies over multiple years could provide important insights if the relative contributions of starvation versus predation to overall mortality rates fluctuates across years, which would be expected under variable food and/or predator abundances. Using a multi-trait, multi-year approach to study risk-taking decisions will allow for a more holistic understanding of the mechanisms shaping these behavioural decisions, which is necessary to understand the consequences of variation in these behaviours on survival.

## Data Availability

All data and code required to reproduce the results and figure in this manuscript are available on the Open Science Framework digital repository: https://osf.io/p3hz4/. Electronic supplemental material is available online [42].
